# Our Profession1Read at the meeting of the Missouri Valley Veterinary Medical Association, October, 1896.

**Published:** 1896-12

**Authors:** F. W. Hopkins

**Affiliations:** Kansas City, Mo.


### OUR PROFESSION.[1]

By F. W. HOPKINS, D.V.S.,
KANSAS CITY, MO.

I HAVE not been able to do justice to this subject, which occurs to me is of such importance to us as veterinarians, and which I have taken the liberty to entitle "Our Profession."

A little more than a decade ago "horse-doctors," as we were wont to be called, were few in number, straggling along the Eastern coast of this country endeavoring to administer to the ailments of the noblest quadruped that treads the earth, and that, too, under most trying circumstances, from the fact that we were surrounded by the d—— rascals (you will pardon the adjective) probably the world produces. From these surroundings our profession has risen, Phœnix-like, to be to-day a part of the bulwarks of this great nation.

In the legislative halls of this government you will find, from the reports of the Department of Agriculture to Congress, that our profession is represented, and that it has advanced from a seeming unimportant branch of that department of government to be to-day in its foremost ranks.

Our profession, from a financial point of view, has been the means of saving this country from a disease that has cost the British government millions of dollars merely to keep under control—in the eradication of that dreaded disease, contagious pleuro-pneumonia, from among our cattle; while through our instrumentality laws have been enacted and a successful quarantine system established, which has probably saved to the people many millions, by placing a check on the indiscriminate entry into our country of cattle from Europe, carrying the contagious plagues with which you are all familiar.

The beneficial influence that is now being so universally felt by the laity as a result of amending our laws, requiring that in future all inspectors in the Bureau of Animal Industry, especially of meat, must hereafter be veterinarians and not only M.D.'s, has brought about a change that has awakened our people to a realization of how essential to their welfare are veterinarians; and to-day its effects are being felt from the

[1] Read at the meeting of the Missouri Valley Veterinary Medical Association, October, 1896.

coast of Maine to California, from Canada to the Gulf, and even in Europe.

From a few veterinarians in the extreme Eastern States we have grown to be an army numbering some five or six thousand men, scattered all over the country. While the majority enjoy the benefits of an extensive practice and are independent, there are a few failures, the result probably of the prevailing hard times and of a deficiency in the executive ability so essential to success, and not from a lack of proficiency. Especially do we find these failures among young practitioners who have been often overcome by their surroundings—the contaminating class of people we as veterinarians are thrown among, and the many indignities to which we are subjected from time to time in going the rounds of an every-day practice. It reminds me of what one of my old quiz-masters at college used to tell us when he said: "Boys, you will often have more trouble in diagnosing the boss than you will the horse." You have all met that animal, which is often so humiliating to the young practitioner, with his wonderful bot-cure and his sure-shot remedy for the fis-tel-o. With such nonsense to annoy him, quite often he is driven to ask himself, What am I anyhow? and he will probably place a valuation on his services that will not afford him a living, and which, I am sorry to say, sometimes ends in collapse and failure. Why, I have heard of qualified veterinarians whose education had cost not less than two thousand dollars, speak of making professional calls for a fee of fifty cents! Just think of it! Now is it to be wondered at that some are starving to death in practice? This is not the result of an overcrowded field or a glutting of the country with veterinarians. No; but, as my quiz-master would say, they have totally failed in diagnosing the boss.

These humiliations have been productive of good as well as bad results, as I think they have done much toward engendering that friendly feeling among us that makes it a relief and a pleasure to be at any time among the fraternity. While some may tell him it is the result of deficient theoretical knowledge when he has failed to trace to its origin the echonorhynchus-jagus or the slepenurd dentata of his theoretical colleague, the young practitioner may retort that "big fleas have lesser fleas on their backs to bite them, while these have little fleas *ad infinitum*."

Now to sum up our profession, we must have the theoretical as well as the practical veterinarian—one is at sea without the assistance of the other. It was through the standing shoulder to shoulder of our old curators, some of whom were practical, others theoretical, that has placed our profession where it stands to-day. They studied your interests in the past; they are doing the same to-day in endeavoring to raise the standard of our profession, so that it may in the not far-distant future take the stand I think it is evidently destined to take—that of the foremost rank in the science of medicine.

We are only in the infancy of our existence, so to speak. The offices we as experts in the diseases of animals will be called upon to fill in civil, military, and national affairs are many, and to-day only commencing to be created. Let us, therefore, qualify, so that selection for the position we are called upon to fill will be because of exceptional ability, and not because of fitness to toot a horn in the band-wagon of national affairs, if I may so express it.

